# Crossing the Old Local Breed Deutsches Lachshuhn with the Layer Breed White Rock: Effects on Laying Performance of the Females and Fattening Performance of the Males

**DOI:** 10.3390/ani13192999

**Published:** 2023-09-22

**Authors:** Daniela Werner, Ralf Bussemas, Lisa Baldinger

**Affiliations:** Thünen Institute of Organic Farming, Trenthorst 32, 23847 Westerau, Germany

**Keywords:** dual-purpose, organic agriculture, Lachshuhn

## Abstract

**Simple Summary:**

For the production of chicken meat and eggs, specialized chicken genotypes are almost exclusively used today. Males of layer lines do not have a satisfactory fattening performance and are usually killed immediately after hatching. Due to ethical concerns, this practice has recently been forbidden in Germany and France and alternatives have to be implemented. One such approach is the determination of the embryos’ sex while in the egg, and keeping only those which contain female embryos. Another alternative is the use of dual-purpose genotypes, where the females can be used for laying and the males for fattening. In this trial the suitability of an old dual-purpose breed (Deutsches Lachshuhn) and its cross with a layer breed (White Rock) for organic egg and meat production was compared to two already established dual-purpose genotypes. Fattening and laying performance of the cross of Deutsches Lachshuhn and White Rock was comparable to that of the other dual-purpose genotypes. This opens the possibility to conserve the genetic resources of an old breed and use them to produce crosses that have a good production performance.

**Abstract:**

We tested the novel cross of the old local breed Deutsches Lachshuhn and the layer breed White Rock, as well as purebred Deutsches Lachshuhn, for their suitability as dual-purpose chickens under 100% organic husbandry conditions, and compared their performance and welfare with the two dual-purpose crosses New Hampshire × Bresse and Bresse × White Rock, which are already established in Germany. Chicks were reared in mixed-sex groups until slaughter of the males at 15 or 18 weeks of life. Data on laying performance and animal welfare were recorded until the hens’ 72nd week of life. Laying performance of Deutsches Lachshuhn × White Rock was almost twice as high as that in purebred hens, while fattening performance of the males did not differ. Deutsches Lachshuhn × White Rock, New Hampshire × Bresse and Bresse × White Rock realized a balanced performance profile of 242–250 eggs per hen alive and a final live weight of the males of 2924–3105 g after 18 weeks of rearing. The efficiency of a pair of chickens (one male and one female) was very similar for the crosses (3.69–3.77 kg feed kg^−1^ marketable product), while purebred Deutsches Lachshuhn was less efficient (6.35 kg feed kg^−1^ marketable product). Crossing the breed Deutsches Lachshuhn with a layer breed therefore improved laying performance and overall efficiency of the birds compared to purebred Deutsches Lachshuhn.

## 1. Introduction

The commercialization of chicken production, which led to the partition of production into egg and meat production, resulted in highly specialized chicken genotypes. While until the mid-20th century a multitude of actors kept and bred a wide variety of chicken genotypes for simultaneous small-scale egg and meat production purposes [1,2], in 2015 the majority of the world’s commercially kept chickens stemmed from only six breeding companies [3]. 

In highly productive laying chicken genotypes, the genetic focus on egg production was accompanied by inferior fattening performance of the males, which renders the use of males from layer lines for the production of chicken meat uneconomical and supposedly environmentally inefficient [4,5,6]. The culling of day-old males of layer lines is commonly undertaken globally to bypass this, but rising ethical concerns about this practice have led to a ban of the killing of day-old males in Germany and France since the beginning of 2022 and 2023, respectively [7,8].

Depending on the type of agricultural management, three alternatives to the culling of males of layer lines are practiced in Germany. In ovo sexing results in hatching of eggs with female embryos only, while eggs with male embryos are treated as a byproduct which can be further processed as animal feed, for instance [6]. In ovo sexing however, is rejected by the leading German associations for organic farming for ethical reasons, as it is nonetheless associated with the unreasonable killing of an animal. Another alternative is the fattening of the males from layer lines. Due to the lower biological productivity and differing proportions of valuable cuts of these animals, an economic cross-compensation in form of higher egg prices and adjusted marketing strategies for the carcasses have to be implemented to reach cost effectiveness [9]. To avoid the ethical dilemma of in ovo sexing and the uneconomical production of chicken meat with males from layer lines, the use of dual-purpose chicken breeds is promoted, especially in the organic farming sector. Compared to specialized hybrid genotypes, the negative environmental impacts of chicken meat and egg production when using either dual-purpose chicken breeds or males of layer lines are higher [10,11]. However, improving the performance of dual-purpose genotypes through breeding could be a compromise between ethical and environmental constraints on chicken egg and meat production.

As a consequence of the specialization and concentration of the chicken breeding industry, most of the known (domestic and traditional) dual-purpose breeds have been kept in a hobby breeding context during recent decades, which focused on external features while neglecting performance. To improve performance, different breeding approaches are possible. While purebred breeding concentrates on selecting animals for performance and health parameters within one breed, crossbreeding aims to combine production traits of different specialized breeds. The crosses of the meat breed Bresse and the layer breeds White Rock and New Hampshire, which were developed by Ökologische Tierzucht gGmbH (ÖTZ), a breeding organization founded by the organic farming associations Bioland and Demeter, are examples of combining both production aims in one breed. Crossbreeding with domestic and traditional breeds has the potential to improve the performance of the resulting crosses while preserving genetic diversity [1].

The Deutsches Lachshuhn is an old local breed and is well suited for free-range management systems due to its low tendency to fly. However, the laying performance is only a maximum of 150 eggs per year [12]. By using it as a sire line in a cross with White Rock, the resulting crossbred hens can be expected to have an improved laying performance. The White Rock chicken is a brown laying breed and White Rock hens from ÖTZ have a laying performance of 225 to 230 eggs (per hen in 294 days of life [13]). They are primarily kept as parents for the production of layer hybrids. 

The aim of the presented study was to test a novel cross of the local breed Deutsches Lachshuhn (Salmon Chicken, translated to German) and White Rock, as well as purebred Deutsches Lachshuhn for their suitability as a dual-purpose chicken under 100% organic conditions. To compare the performance of Deutsches Lachshuhn and its crosses with White Rock to already established dual-purpose crosses, the above mentioned ÖTZ crosses of New Hampshire × Bresse and Bresse × White Rock were also included in the study.

## 2. Materials and Methods 

### 2.1. Animals and Experimental Design

In this study, the performance of the following three crosses and one pure bred genotype were compared:

- Deutsches Lachshuhn (LH);
- ♂ Deutsches Lachshuhn × ♀ White Rock (LH × WR);
- ♂ New Hampshire × ♀ ÖTZ Bresse (NH × Bresse);
- ♂ ÖTZ Bresse × ♀ White Rock (Bresse × WR).

The performance of crosses of Bresse and NH or WR was tested in a previous study [14,15] and the crosses have since been made available to the public under the names ÖTZ Coffee and ÖTZ Cream. To further monitor the development of these fairly new crosses, they were again included in this study.

The parent flocks for NH × Bresse and Bresse × WR were kept at Geflügelhof Bodden (47574 Goch-Hommersum, North Rhine-Westphalia). The LH parent flock and their crosses with WR were kept at the farm of LH breeder and project partner Wendland Geflügel (29496 Waddeweitz, Lower Saxony), where 120 to 160 LH breeding hens and 6 LH rooster lines were available. Hatching took place at the hatchery of Wendland Geflügel, and the incubation started at the 2 July 2019. A total of 885 chicks hatched on the 23 July 2019. The number of hatching eggs and hatched chicks per genotype is listed in Table 1; a description of the data set can be found in Table S1 in the Supplemental Materials.

**Figure 1:**
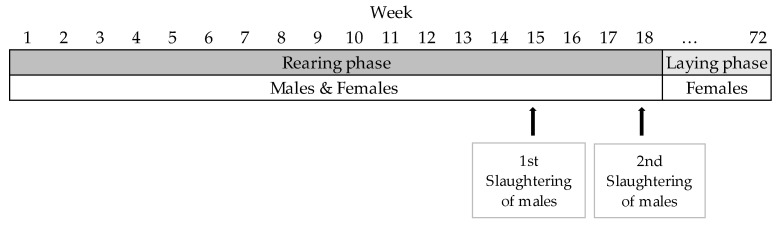
Phases of the study.

To compare the fatting performance of the males and the animal welfare of the four genotypes, male and female chicks were reared in two mixed-sex groups per genotype until slaughter of the males at an age of 15 and 18 weeks. To compare the laying performance and animal welfare of the four genotypes, females were then kept for one laying period until their 72nd week of life. Figure 1 shows the phases of the study.

Pullets and males were submitted to the same vaccination scheme against Mareks disease, infectious bronchitis, Salmonella, coccidiosis, infectious bursal disease, Newcastle disease, laryngotracheitis, avian encephalomyelitis, and Mycoplasma, except for one vaccination via injection in week 17 (avian rhinotracheitis, infectious bronchitis, Newcastle disease, egg drop syndrome) for the pullets only.

### 2.2. Housing

#### 2.2.1. Rearing Period

Pullets and males were reared from 23 July to 25 November 2019.

After hatching, one mixed-sex group of day-old chicks per genotype (e.g., four groups) was transferred to one of two mobile barns (a customized version of model Regio, Wördekemper GmbH & Co. KG, 33397 Rietberg, North Rhine-Westphalia, Germany, measuring 8 × 4 m). At an age of 4.5 weeks, groups were split into two groups per genotype, consisting of 45 pullets and 45 males per group.

Each mobile barn was customized to provide space for four compartments of 7.5–8.0 m^2^, sufficient for 200 pullets or 42 laying hens each. The compartments were equipped with infrared gas-powered radiant heaters, drinkers, troughs, and wooden perches. Chopped straw and spelt husks were used as bedding material. A netted green outdoor area with a space of 4 m^2^ per animal was available for each group and access to the outdoor run was granted from the age of 6 weeks onwards.

#### 2.2.2. Laying Period

After the slaughter of the males, two groups of pullets of each genotype (40–43 animals per group) with 2 males each remained in the mobile barns. Barns were equipped with group nests, drinkers, and two round feed troughs per group. Pick blocks and grit were available. Chickens had access to a green outdoor area of 4 m^2^ per animal from 10 AM until dusk.

### 2.3. Feeding

Feeding during the rearing period was the same for all genotypes and was divided into four feeding phases. The laying period was divided into two feeding phases. All feed mixtures were purchased from a commercial feed mill, consisting of 100% organically produced components, and were ground, except for Starter 1 and Layer mixtures which were granulated. During the laying period and starting in week 24, hens additionally received a quantity of whole grains of 10% of the daily feed consumption, which was adjusted once a week. The analyzed nutrient composition of the feed mixtures can be found in Table 2. Throughout the study, feed was supplied ad libitum.

### 2.4. Data Collection

#### 2.4.1. Rearing Period

During the rearing period, feed consumption and live weight were documented every three weeks. To provide data on slaughter performance of the males at different ages, 30 males of each genotype were slaughtered at an age of 15 weeks, and the remaining males were slaughtered at an age of 18 weeks. Animal losses were documented continually. Data on animal welfare were collected at week 12 and 18 for pullets and males, and at week 12 and 15 for earlier slaughtered males, following a simplified version of the Welfare Quality Protocol [16], with a score of “0” for uninjured, “1” for minor injuries, and “2” for major injuries. The occurrence of breast blisters was only assessed for males, and deformations of the keel bone only for pullets. Signs of laying activity were classified into three categories (0 = yes, 1 = maybe, 2 = no).

Final live weight and carcass weight were used to calculate carcass yield, and valuable cuts (breast fillet, legs) of three males per genotype were weighed. The three males per genotype were selected to represent the mean final live weight ± one SD for each genotype.

One sample of each feed mixture during rearing was sent to a commercial laboratory and analyzed for nutrients according to the European Commission Regulation EC 152/2009 [17].

#### 2.4.2. Laying Period

During the laying period, feed consumption was documented on a weekly basis, and live weight of the hens was recorded at weeks 24, 32, 38, 50, 59, and 72. Animal welfare was assessed at weeks 50 and 72 according to M-Tool [18]. Data on laying performance were collected daily per group and included the total number of eggs, the number of marketable eggs, and the size distribution. Eggs were classified as non-marketable when they were found on the floor, dirty, cracked, or without shells. Egg quality, including the egg shape index, which was calculated via caliper measurements of the height and width of the eggs, was recorded on a group basis at weeks 24, 32, 40, 56, and 72 and was based on a sample of three eggs of each size class (size classes based on the weight according to market rules) from each genotype and both hen groups. The laying period ended with the slaughter of the hens at the age of 72 weeks, and valuable cuts (breast fillet, legs) of three hens per genotype (only two for LH × WR) were weighed. 

One sample of each feed mixture during laying was sent to a commercial laboratory and analyzed for nutrients according to the European Commission Regulation EC 152/2009 [17].

### 2.5. Statistical Analysis

Statistical analyses were conducted with proc glimmix (SAS 9.4). *p*-values < 0.05 were considered to indicate significant differences. Only data of birds which were weighed at least twice were included in the analysis of live weight and daily weight gain (only males).

#### 2.5.1. Rearing Period

The model for analyzing live weight and daily weight gain (only males) included the fixed effects of breed (k = NH × Bresse, Bresse × WR, LH, LH × WR), age in weeks (l = 3, 6, 9, 12, 15, 18), the interaction of breed and age, and the random effect of bird within breed:

Y = µ + breed_k_ + week_l_ + breed_k_ × week_l_ + bird(breed) + ε

For the analyses of feed consumption and feed conversion ratio, the model described above was used excluding the random effect of the animal as data on these traits were measured on a group basis. Multiple comparisons of means were conducted using the Tukey test. Due to the low sample size, data on valuable cuts are presented descriptively.

As data on slaughter performance and animal welfare were analyzed separately for each observation date, the model only included the fixed effect of the genotype. Multiple comparisons of means for slaughter performance were conducted via the Tukey test. The relative frequency of the scores of animal welfare assessments for each observation date was analyzed using a chi^2^ test with a multinomial distribution. *p*-values of multiple comparisons of means were adjusted according to Bonferroni–Holm. 

#### 2.5.2. Laying Period

The model for analyzing live weight included the fixed effects of breed (k = NH × Bresse, Bresse × WR, LH, LH × WR), age in weeks (l = 18, 24, 32, 38, 50, 59, 72), the interaction of breed and age, and the random effect of bird within breed:

Y = µ + breed_k_ + week_l_ + breed_k_ × week_l_ + bird(breed) + ε

Due to the low sample size, data on slaughter performance are presented descriptively. 

Animal welfare indicators were analyzed separately for each observation date (weeks 18, 50, 72); hence, the model only included the fixed effect of the breed. Multiple comparisons of mean for slaughter performance were conducted via the Tukey test. The relative frequency of the respective scores in the animal welfare assessment for each observation date was analyzed using a chi^2^ test with a multinomial distribution, and *p*-values of multiple comparisons of means were adjusted according to Bonferroni–Holm.

### 2.6. Ethics Approval

Animal husbandry of parent flocks and crosses followed the rules of the European Council Regulation EC 2018/848 [19]. Both the rearing and laying period took place at Wendland Geflügel (29496 Waddeweitz, Lower Saxony, Germany). Because all data collection was part of the common practices of animal husbandry and was performed by the farmer Sebastian Seelig, the study did not fall under the category “animal experiment”, and therefore no announcement at the directorate for animal protection at the ministry in Lower Saxony was necessary.

## 3. Results

### 3.1. Rearing Period

#### 3.1.1. Feed Consumption and Feed Conversion Ratio during Rearing

Because pullets and males were reared together, feed consumption during rearing refers to mixed-sex groups of birds. Feed consumption did not differ between the breeds and was 72–80 g day^−1^ until the first slaughter date after 15 weeks, and 78–85 g day^−1^ until the second slaughter date after 18 weeks (Table 3). Accordingly, the feed conversion ratio also refers to the rearing of mixed-sex birds, and was equally not affected by breed. Feed conversion ratio was 3.5–3.6 kg feed kg^−1^ weight gain until the age of 15 weeks, and 4.0–4.1 kg feed kg^−1^ weight gain until the age of 18 weeks.

#### 3.1.2. Fattening and Slaughter Performance of the Males

**Figure 2:**
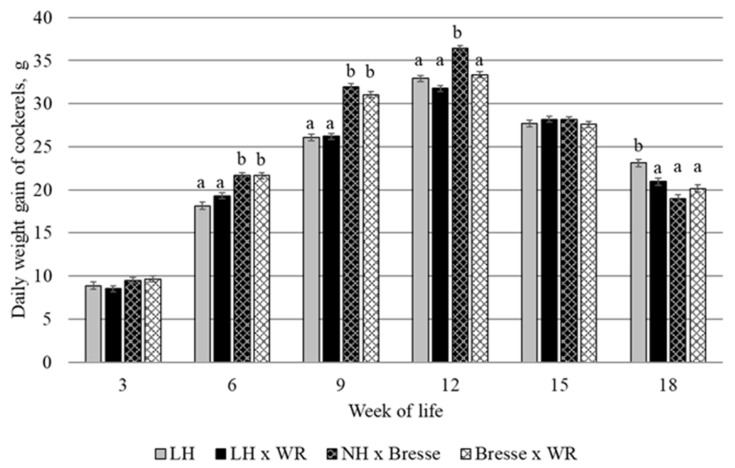
Daily weight gain of the males of dual-purpose breeds (LH = Deutsches Lachshuhn, WR = White Rock, NH = New Hampshire); lsmeans of the interaction GT × age; columns with no letter in common indicate significant differences.

For both slaughter dates, there was a significant interaction between breed and age for live weight and DWG (Table 3), with LH and LH × WR males reaching 12 and 8% lower final live weights than NH × Bresse and Bresse × WR, while LH and LH × WR did not differ from each other. As shown in Figure 2, DWG of all breeds was highest in week 9–12 and ranged from 31.8 to 36.4 g. In week 3–9, DWG of LH and LH × WR did not differ from each other and was lower than that in NH × Bresse and Bresse × WR, which also did not differ from each other. In week 9–12, DWG of NH × Bresse males was still highest and was 9.0–14.5% higher than that in the other breeds, which did not differ. In week 12–15, there was no longer a difference between the breeds, and in week 15–18, DWG was 22 and 15% higher in LH than in NH × Bresse and Bresse × WR, with LH × WR between the two. Carcass weight and dressing percentage of LH and LH × WR did not differ from each other and were lower than those in NH × Bresse and Bresse × WR males at both slaughter dates. 

NH × Bresse and Bresse × WR differed insofar as NH × Bresse males had a higher carcass weight after 15 weeks and a higher dressing percentage after 18 weeks than their Bresse × WR counterparts. Three males per breed and slaughter date were cut up for valuable parts, which revealed that after 15 weeks breast fillet proportion was highest in Bresse × WR, and after 18 weeks was highest in NH × Bresse. The proportion of leg from the total carcass did not differ between the breeds and was 39.0–39.5% after 15 weeks and 39.4–42.9% after 18 weeks. 

#### 3.1.3. Animal Welfare of the Males

**Figure 3:**
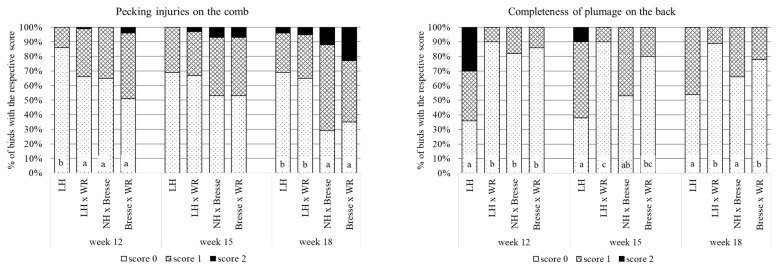
Pecking injuries on the comb and completeness of the plumage on the back of the males of dual-purpose breeds; % of birds with the respective score, with a score of “0” indicating an unimpaired state, “1” minor changes and “2” major damages (LH = Deutsches Lachshuhn, WR = White Rock, NH = New Hampshire; columns with no letter in common indicate significant differences).

Analysis of the welfare assessments of the males revealed significant differences between the breeds for pecking injuries on the combs and for completeness of the plumage on the back; see Figure 3. The highest frequency of intact combs (score “0”) was found in LH males, both in week 12 and 18. LH × WR males had a lower frequency of score “0” in week 12, but did not differ from LH in week 18. NH × Bresse and Bresse × WR did not differ from each other, and in week 15 the four breeds did not differ at all. When the males were 12 weeks old, the plumage on the back was least complete (=lowest frequency of score “0”) in LH, while the other breeds did not differ. At 15 weeks, plumage on the back was less complete in LH than in LH × WR, with NH × Bresse and Bresse × WR between the two. At both 12 and 15 weeks, a score of “2” was only given to LH males. At 18 weeks, none of the males received a score of “2”, but the plumage on the back of LH and NH × Bresse was less complete than that in LH × WR and Bresse × WR.

Other noteworthy observations regarding welfare were as follows: No hock burns were found on any of the males, and only a single Bresse × WR male showed a foot pad lesion at the age of 15 weeks. Breast blisters were found at all ages and, without a difference, in all breeds: the frequency of score “0” was 91–99% at 12 weeks, 83–97% at 15 weeks, and 75–86% at 18 weeks of age. Pecking injuries on the back were rarely found, and were also not influenced by breed: the frequency of males without pecking injuries on the back was 96–100% at 12 weeks, 90–100% at 15 weeks, and 91–100% at 18 weeks of age. For additional welfare indicators not described here, see Table S2 in the Supplemental Materials.

### 3.2. Laying Period

#### 3.2.1. Feed Consumption during Laying

Consumption of the feed mixture (“Layer 1” and “Layer 2” as shown in Table 2) was both influenced by the breed and the age of the laying hens. Averaged over the whole laying period, LH hens consumed the least feed (111 g), while the other breeds did not differ and consumed 122–125 g feed day^−1^. The amount of 10% of the total feed consumption of whole oat grains has to be considered additionally to feed intake during laying, but cannot be quantified exactly as the true intake could not be documented due to the experimental setup.

#### 3.2.2. Laying Performance and Efficiency of the Hens

**Figure 4:**
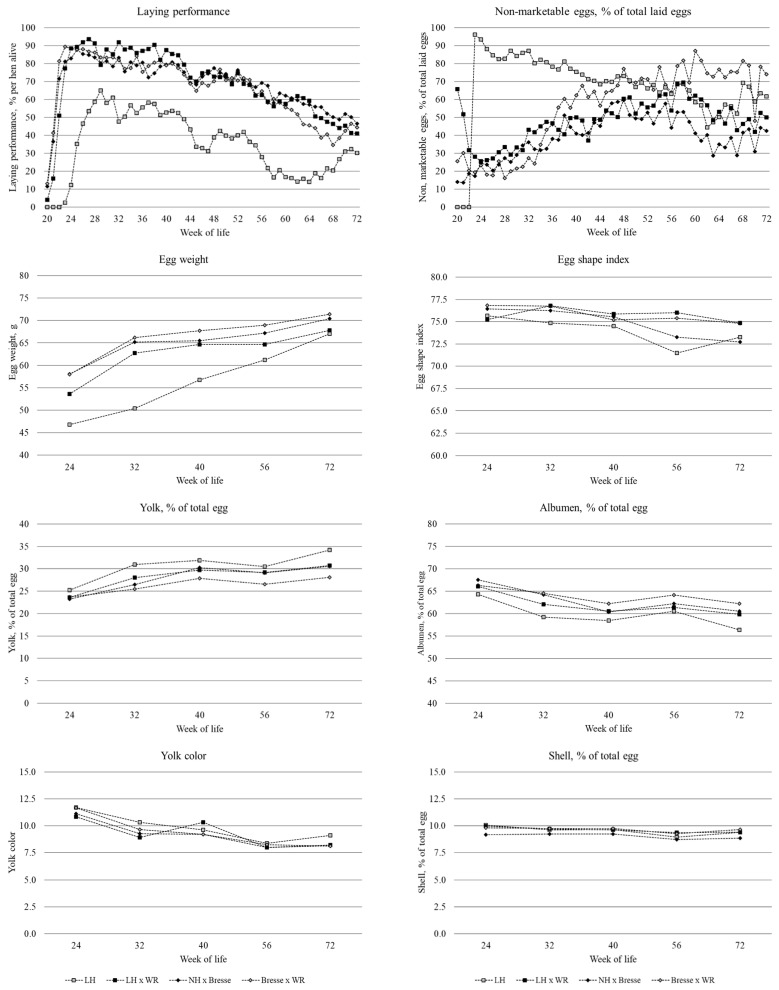
Laying performance (% per hen alive), egg composition and yolk color (assessed with a DSM YolkFan^TM^ with a range of 1–15) of the hens of dual-purpose breeds (LH = Deutsches Lachshuhn, WR = White Rock, NH = New Hampshire); least square means with no letter in common indicate significant differences.

While LH hens had a consistently lower laying performance, the laying curve as depicted in Figure 4 was very similar in LH × WR, NH × Bresse, Bresse × WR, and LH × WR hens. Averaged over the whole laying period, the laying performance per hen alive was lower in LH hens (34.7%) but did not differ between LH × WR, NH × Bresse, and Bresse × WR (66.4–68.8%, Table 4). 

The sum of total laid eggs per hen alive was 126 in LH hens and 249, 242, and 250 in NH × Bresse, Bresse × WR, and LH × WR hens. This translated to a total egg mass produced by an average hen alive of 6.9 kg in LH hens and 16.3, 16.1, and 15.7 kg in NH × Bresse, Bresse × WR, and LH × WR, respectively. The feed conversion ratio for laying was 5.90 kg feed kg^−1^ weight gain for LH hens, 2.92 for LH × WR hens, 2.78 for NH × Bresse hens, and 2.92 for Bresse × WR hens. Peak production was 64.9% in week 29 for LH hens, 93.5% in week 27 for LH × WR hens, 87.6% in week 25 for NH × Bresse hens, and 89.4% in week 23 for Bresse × WR hens. The onset of lay (= 10% laying performance) was in week 24 for LH and in weeks 21, 20, and 20 for LH × WR, NH × Bresse, and Bresse × WR hens, respectively. The proportion of non-marketable eggs of total eggs is shown in Figure 4. In LH hens, the proportion of non-marketable eggs was almost 100% at the beginning of lay, and thereafter decreased to around 60% at week 72. In LH × WR, NH × Bresse, and Bresse × WR, it started to increase after week 32, reached a level of 50–70% at the age of 44–56 weeks, and after that decreased in LH × WR and NH × Bresse until the end of the laying period, but stayed at a level of around 70–80% in Bresse × WR. The sum of marketable eggs per hen alive was 35 for LH hens and 133, 152, and 115 for LH × WR, NH × Bresse, and Bresse × WR hens, respectively.

#### 3.2.3. Egg Quality

Egg weight was influenced by breed and age, and average egg weight was highest in Bresse × WR, followed by NH × Bresse, LH × WR, and LH (Table 4). Over the course of the laying period, egg weight increased in all breeds, starting with an egg weight of 47–58 g in week 24 and reaching 67–71 g in week 72 (Figure 4). Of all laid eggs, the respective proportion of small, medium, large, and extra-large eggs was 14.2, 65.8, 18.0, and 1.2% in LH; 5.0, 43.0, 41.7, and 9.8% in LH × WR; 3.2, 28.8, 45.2, and 21.7% in NH × Bresse; and 4.3, 34.2, 45.5, and 15.3% in Bresse × WR. Only very few eggs were classified as extra-small; these made up 0.8, 0.5, 0.6, and 0.7% of all eggs of LH, LH × WR, NH × Bresse, and Bresse × WR, respectively. The egg shape index was influenced by breed and age, and was lower in LH than in LH × WR and Bresse × WR, while NH × Bresse eggs did not differ from the others. In week 24, egg shape index was 75–77, which slightly decreased over time to 73–75 in week 72. In all breeds, yolk percentage increased and albumen percentage decreased over time. However, a significant interaction between breed and age was found for yolk percentage. While there was no difference in yolk percentage at the age of 24 weeks (23.6–25.2%), for the rest of the laying period, yolk percentage of LH was always higher than that in Bresse × WR eggs, with yolk percentages reaching 28.1–34.2% at the end of the laying period. Yolk percentage in LH × WR and NH × Bresse eggs never differed from that in Bresse × WR eggs, and most of the time (weeks 32, 40, and 56) also did not differ from that in LH eggs. Albumen percentage, on the other hand, was lower in LH than in Bresse × WR eggs, with LH × WR and NH × Bresse between the two. In week 24, albumen percentage was 64.3–67.5%, which decreased to 56.4–62.2% in week 72. Shell percentage was influenced by breed and age, and was lowest in NH × Bresse eggs, while the other breeds did not differ from each other. From week 24 to 72, shell percentage decreased slightly from 9.2–10.0 to 8.9–9.4%. For yolk color, a significant interaction between breed and age was found, but pairwise comparisons only revealed a tendency towards a darker yolk in LH eggs than in LH × WR eggs in week 32. Averaged over the whole laying period, yolk was darker in LH eggs than in LH × WR and NH × Bresse eggs, with Bresse × WR between the two and not differing from the others. Yolk color slightly decreased over time from 10.8–11.7 in week 24 to 8.2–9.1 in week 72.

#### 3.2.4. Live Weight Development and Slaughter Performance of the Hens

At the end of rearing, LH × WR pullets were 7% lighter than NH × Bresse, with LH and Bresse × WR pullets between the two (Table 4). During the laying period, a significant interaction of breed × age was found for live weight of the hens (Table 4). LH and LH × WR hens slightly lost weight from week 32 to week 50, and only gained from week 50 to week 72. On the other hand, both NH × Bresse and Bresse × WR hens increased their live weight from 32 to 50 to 72 weeks. At all weighing dates throughout the laying period, LH hens were the heaviest, and Bresse × WR hens the lightest birds, with final live weights of LH hens being 16% higher than those in Bresse × WR. Live weights of LH × WR and NH × Bresse hens were always between those of LH and Bresse × WR. After slaughter of the senior hens at the age of 72 weeks, three carcasses per breed (only two of LH × WR) were weighed and cut up for valuable parts (Table 4). Both final live weight and carcass weight were highest in the sample of LH hens, while all other breeds did not differ. Dressing percentage was highest in LH and lowest in Bresse × WR, with LW × WR and NH × Bresse not differing from the others. The proportion of breast fillet and leg of total carcass was not influenced by breed and was 17.0–18.8 and 30.0–33.7%, respectively. 

#### 3.2.5. Animal Welfare of the Laying Hens

**Figure 5:**
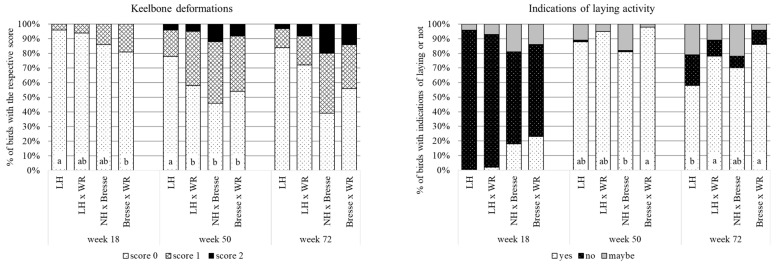
Keel bone deformations (score of “0” indicating an unimpaired state, “1” minor changes, and “2” major damages) and indications of laying activity (“yes”, “no”, and “maybe”) of the hens of dual-purpose breeds; % of birds with the respective score (LH = Deutsches Lachshuhn, WR = White Rock, NH = New Hampshire; columns with no letter in common indicate significant differences).

Analyses of the welfare assessments during the laying period revealed significant differences between the breeds regarding keel bone deformations and laying activity, as shown in Figure 5. While none of the hens had a major deformation or fracture of the keel bone (score “2”) at 18 weeks, scores of “2” appeared in all breeds at 50 and 72 weeks. At 18 weeks, the frequency of hens with intact keel bones was highest in LH (96% score “0”) and lowest in Bresse × WR hens (81%), with LH × WR and NH × Bresse between the two (86 and 94%, respectively). At 50 weeks, again the most hens with intact keel bones were found in LH (78% score of “0”), while the others did not differ from each other (46–58%). At 72 weeks, there was no difference between the breeds, and the frequency of intact keel bones ranged from 39 to 84%. With regard to laying activity, irrespective of breed, most hens did not show signs of laying at 18 weeks (63–91% score of “no”). At 50 weeks, most hens showed signs of laying, with the highest frequency in Bresse × WR (98% score of “yes”) and the lowest in NH × Bresse (81%), with LH and LH × WR between the two and not differing from the others (88 and 95%, respectively). At 72 weeks, the frequency of hens with signs of laying was lower in LH (58%) than in LH × WR and Bresse × WR (78 and 86 score of “yes”, respectively), with NH × Bresse (70%) not differing from the others.

Other noteworthy observations regarding welfare were as follows: No foot pad lesions were found on any of the hens at 18 weeks. At 50 weeks, however, footpad lesions were documented in all breeds, and the frequency of score “1” ranged from 2 to 11%, without being influenced by breed. At 72 weeks, the frequency of hens with footpad lesions was lowest in LH hens (3% score of “1”), while the other breeds did not differ and 17–23% of the hens received a score of “1”. A score of “2” was never documented. The plumage around the cloaca was complete in all hens at 18 weeks, but at week 50, the breeds differed significantly: while only 43% of Bresse × WR hens had a complete plumage, 95–97% of the LH, LH × WR, and NH × Bresse hens received a score of “0”. At 72 weeks, the completeness of plumage around the cloaca was not influenced by breed, with values ranging between 100% (LH and LH × WR) to 96% (NH × Bresse) and 38% (Bresse × WR). Pecking injuries around the cloaca were found in only one single NH × Bresse hen at the age of 18 weeks, but were more frequent in week 50 and 72. While in LH, LH × WR, and NH × Bresse, only one hen per breed showed a pecking injury at the age of 50 weeks, the frequency of score “1” was much higher than that in Bresse × WR hens (23%). At 72 weeks, the frequency of pecking injuries around the cloaca was not influenced by breed, with no injuries at all in LH and LH × WR hens and values ranging from 4% score of “1” in NH × Bresse to 14% score of “1 and 2” in Bresse × WR. For additional welfare indicators not described here, see Table S3 in the Supplemental Materials.

#### 3.2.6. Sibling Performance

Table 5 summarizes the feed consumption during rearing and laying, as well as the carcass weights for males and females and the total produced egg mass. The feed conversion ratio of a pair of siblings expressed as the sum of consumed feed divided by the sum of marketable product was 6.35 in LH birds and ranged from 3.69 to 3.77 in the breeds LH × WR, NH × Bresse, and Bresse × WR.

#### 3.2.7. Animal Losses

During the first 6 weeks of mixed-sex rearing, animal losses in LH were 12.2% of the initial 226 birds, while in LH × WR, NH × Bresse, and Bresse × WR, losses of 2.7, 2.0, and 1.7% of the initial 278, 203, and 178 birds were documented. From week 7–18, another 3.3, 3.3, 0.6, and 1.1% of LH, LH × WR, NH × Bresse, and Bresse × WR birds were lost. During the laying period, animal losses amounted to 18 hens out of the initial 330. Reasons for losses were culling because of injuries (2 hens) and cloaca prolapses (2 hens), and unknown reasons (12 hens). Expressed as a percentage of the initial numbers of hens, losses were 7.2, 1.2, 3.6, and 9.9% in LH, LH × WR, NH × Bresse, and Bresse × WR hens, respectively.

## 4. Discussion

### 4.1. Fattening and Slaughter Performance of the Males

Half of the males of the dual-purpose breeds were slaughtered at the age of 15 weeks and the other half at the age of 18 weeks. At both slaughter dates, final live weight, daily weight gain from hatching to slaughter, carcass weight, and dressing percentage were lower in LH and LH × WR than in NH × Bresse and Bresse × WR (Table 3). The fattening performance of purebred LH and the novel cross LH × WR can therefore be regarded as inferior to the already established dual-purpose crosses NH × Bresse and Bresse × WR. However, a closer look at daily weight gain throughout the fattening period reveals interesting changes over time (see Figure 2): while daily weight gain of LH and LH × WR was lower than that in NH × Bresse and Bresse × WR until the end of week 9, and still lower than that in NH × Bresse in week 9–12, in week 15–18, daily weight gain was highest in LH. Purebred LH therefore show a flatter growth curve than NH × Bresse and Bresse × WR, which allows the conclusion that LH males should be slaughtered later than the other breeds. The novel cross LH × WR did not differ from LH in any aspect of fattening or slaughter performance, except for daily weight gain in week 15–18, which was higher in LH. Still, all observations indicate that crossing with WR did not impair the fattening performance of the males. 

Information on the fattening performance of purebred LH is scarce. The breeders’ club for Deutsches Lachshuhn gives a live weight of 3–4 kg for fully grown LH roosters [20], which agrees with a performance test of LH layers in which the two accompanying roosters reached a live weight of 3.75 kg at the age of 23 weeks [12]. The average weight of the LH males in our study was only 2944 g, but at the considerably lower age of only 18 weeks, which has to be taken into account. With regard to the fattening performance of NH × Bresse and Bresse × WR, more information is available with which to compare our results. The NH × Bresse and Bresse × WR males in our study had daily weight gains of 24.7–25.5 g until slaughter in week 15, and therefore grew faster than in previous studies reporting daily weight gains of 22.4 g for both crosses until week 15 [14] and of 22.1 g for Bresse × NH until week 16 [21]. The reason for the faster growth in our study most likely lies in the energy concentration of the feed, which from weeks 8 and 7 on, respectively, was higher in our study (12.2 MJ AME_N_) than in Baldinger and Bussemas [14] (11.3 MJ AME_N_), but comparable to that in Lambertz et al. [21] (12.3 MJ AME_N_). Still, the higher growth rate observed in our study indicates that males of NH × Bresse and Bresse × WR could realize their growth potential better under the feeding regime applied here than in the previous regimes. Because the birds were reared in mixed-sex groups in all three studies, further studies on rearing the males separately from the females and with feed adjusted for faster growth might give insight into the real growth potential of these crosses. The feed conversion ratio until slaughter at 18 weeks was 4.0 in LH and 4.1 in all other breeds, which is higher than the values reported by Tiemann et al. [4] for the dual-purpose hybrid Lohmann Dual (FCR 2.32) and the native dual-purpose breed Rhinelander (FCR 3.89) until week 20. Tiemann et al. [4] also initially reared the sexes together, but separated them at the age of 10 weeks and thereafter fattened the males separately, which might explain part of the difference.

Due to the breeding focus on a balanced performance of eggs and meat instead of maximum meat yield, dual-purpose chickens generally show a lower percentage of breast fillet compared to broilers, but an equal or higher percentage of legs from the total carcass. This can again be seen in our study, despite the small sample size of only three males per breed and slaughter date. The percentage of breast fillet recorded in LH and LH × WR (15.1–15.9%) at 15 weeks is in accordance with values reported for purebred White Rock and New Hampshire males at the age of 16 weeks (15.3 and 17.5%, respectively; [22]). Bresse × WR (week 15) and NH × Bresse (week 18) reached higher levels of 17.6 and 19.1%, respectively. The latter are closer to the 20.0% breast fillet reported for the mixed-sex slow-growing broiler Sasso 51 [23], but still lower than the 23.8–24.3% found in the male slow-growing broiler Hubbard JA 757 [24]. Marketing of the males of dual-purpose breeds therefore remains a challenge and cannot be based on premium prices for breast fillet alone. The percentage of legs did not differ between the breeds and was 39.0–39.5% in week 15, which is slightly lower than in a previous study in which we found values of 40–42% [14], but higher than reports for the slow-growing broiler Sasso 51 (32.7%) and the dual-purpose breeds Lohmann Dual (35.8%), Belgian Malines (35.7%), and Schweizerhuhn (33.8%) [23]. 

### 4.2. Animal Welfare of the Cockerels

Male chickens, irrespective of being layer hybrids or dual-purpose breeds, are fattened longer than broilers because of their lower growth rate. Depending on the breed and the length of the fattening period, the males can reach puberty before slaughter, and aggressive behavior can start to appear. As part of the welfare assessment, we documented pecking injuries on the combs of the males as an indicator of aggressive behavior. As expected, the frequency of scores “1” and “2”, indicating minor and major damage, respectively, increased with age, with the highest frequency of a score of “2” (23%) found in Bresse × WR males at the age of 18 weeks. In purebred LH males, on the other hand, no bird received a score of “2” in week 12 and 15, and only 4% of the males were scored “2” in week 18. The novel LH × WR cross showed less intact combs than LH in week 12, but did not differ from LH at the other assessment dates. These observations confirm that the breed LH is very docile, making both the purebreds and the cross good options for farmers worried about aggressive behavior in their males. With regard to plumage, the welfare assessment revealed that some of the purebred LH males were very slow feathering, with 30% of the birds receiving a score of “2” for completeness of the plumage on the back in week 12. In week 18, none of the birds no longer received a score of “2”, indicating that the plumage on the back had finally closed. Crossing LH with WR eliminated this issue, as no score of “2” was documented in any LH × WR male or in any of the other breeds. A welfare issue that was found in all breeds and at all ages was the appearance of breast blisters. In broilers, breast blisters are usually caused by factors such as high stocking density and reduced activity [25], which, in males of dual-purpose breeds kept according to the guidelines or organic agriculture, are unlikely causes. The most likely explanation in our study is the existence of horizontal components in the barn that had quite sharp edges and that birds of all breeds used as perches. This was not our intention, and the components were removed after the study.

### 4.3. Feed Consumption and Laying Performance of the Hens

According to the breeding standard for LH, the breed has an “attractive” laying performance [20]. Detailed data about the laying performance of LH hens are scarce, but in a performance test of LH layers, 132 eggs per hen were achieved [12]. The laying performance of the LH hens in our study was only slightly lower, indicating that they could realize their genetic potential. The laying performance of NH × Bresse and Bresse × WR (68.5% and 66.4%, respectively) was almost exactly the same as that in a previous study, in which a laying performance of 69% and 68% was found, respectively. Refs. [15,21] documented a considerably lower laying performance of 54.2% in Bresse × NH, which can most likely be explained by the use of natural light only, whereas in our study a lighting regime was applied.

The novel cross LH × WR and the two established dual-purpose crosses NH × Bresse and Bresse × WR did not differ in their feed consumption and laying performance expressed in total laid eggs, while both were lower in purebred LH hens. LH × WR hens consumed 9.9% more feed mixture than their LH counterparts, but laid almost double the number of eggs (250 vs. 126 total eggs per hen alive). Crossing with WR therefore considerably improved both laying performance and laying efficiency, with FCR decreasing from 5.6 in LH to 2.9 in LH × WR. The laying curve of LH × WR closely resembled that of NH × Bresse and Bresse × WR, while purebred LH hens started laying later (10% laying performance in week 24 vs. 20–21%), and peaked later (week 29 vs. 23–27) and lower (64.9% vs. 87.6–93.5%) than the other breeds, again underlining the effect of the lack of breeding focus on laying performance in this breed. The concept of crossing an indigenous breed with a layer breed in order to obtain a higher laying performance while providing an incentive to keep the pure breed for genetic diversity is not new, and among other breeds has been shown to be effective in the cross of Vorwerkhuhn × Lohmann layer line [26] and for various crosses of Vorwerkhuhn, Bresse, and White Rock [27].

### 4.4. Egg Shell Quality and Non-Marketable Eggs

While the laying performance expressed in total laid eggs in all breeds was either close to expectations or, in the case of LH × WR, encouragingly higher than in the pure breed, the laying performance expressed in marketable eggs was much lower. At the beginning of lay, almost all eggs laid by LH hens were not marketable because they were laid on the floor. The barn in which the study took place was equipped with group nests mounted on the wall at an unusual height of 1 m (measured without litter), which apparently was too high for the LH hens. Additional nests at a lower height were added and thereafter nest use improved and the proportion of marketable eggs increased. In the other breeds, a few hens also laid their eggs on the floor, as can be seen in the percentage of non-marketable eggs at the beginning of the laying period. However, starting around week 32, the percentage of non-marketable eggs started to increase in LH × WR, NH × Bresse, and Bresse × WR, and most eggs were classified as non-marketable because of inadequate shell strength and a tendency to soil quickly due to a rough shell surface. Calcium formate and vitamin D3 were added to the feed mixture in order to rectify possible calcium deficiencies and the hens were examined for possible illnesses, but the problem persisted. In week 53, the veterinary finally sacrificed two hens of each breed and diagnosed a fatty liver syndrome during the examination. Thereafter, the feed mixture was fortified with choline chloride, which successfully decreased the percentage of non-marketable eggs in LH, LH × WR, and NH × Bresse. Fatty liver syndrome is a non-infectious metabolic disorder in layers with several possible causes, among which is an increased body weight that leads to fat deposition in the liver [28,29]. The role of the liver in egg formation is crucial, and besides the production of nutrients for yolk and albumen, it also produces the proteins for the matrix of the egg shell and is involved in activating vitamin D3 to stimulate calcium absorption for eggshell formation, which explains the negative effect on the egg shells [30,31]. The most likely cause of the fatty liver syndrome of the layers was the energy concentration in the feed during rearing, which was higher than planned from week 8 onward. At 18 weeks of age, the NH × Bresse and Bresse × WR pullets in our study already weighed 2204 g and 2133 g, respectively; these were almost as high as live weights at 20 weeks in a previous study, at 2244 g and 2173 g, respectively [15]. While the cockerels could use the additional energy for improved growth (see there), the pullets apparently could not, and deposited the superfluous energy as fat in the liver. The appearance of fatty liver syndrome can therefore be seen as a confirmation that in mixed-sex rearing of dual-purpose chickens, the focus must be on the pullets, not on the cockerels. As a side note, the farmer who took care of the birds kept the LH × WR layers after the end of the study and let them go through a long molting period of 3 months. During that period, the hens only had natural light, and were fed a feed mixture low in energy and high in fiber (8.5 MJ AME_N_, 112 g crude fiber kg^−1^ feed). The birds laid 208 eggs per hen alive during the ensuing second laying period of 12 months; see laying curve in the Supplemental Materials. The farmer stated that egg shell strength was restored to normal, which might be a sign that the fatty liver syndrome can be cured if layers are kept long enough.

### 4.5. Slaughter Performance of the Hens

In order to improve the total efficiency and economic success of dual-purpose production, not only can and should carcasses from the males be marketed, but also carcasses of the senior laying hens. At the end of the laying period, we found the highest live weight in LH hens, and the lowest in the two WR crosses, with NH × Bresse between the two. Combined with the fact that laying performance was lowest in LH and did not differ between the others, the higher final live weight of LH hens clearly illustrates that this breed has a more pronounced focus on meat performance than the others. The final live weights found in LH × WR, NH × Bresse, and Bresse × WR (2428–2613 g) are lower than previously reported for NH × Bresse and Bresse × WR (2887 and 2944 g, [15]) and Bresse × NH (2857 g, [21]), but reached similar levels as the Italian dual-purpose breed Ermellinata di Rovigo (2609 g, [32]) and Schweizerhuhn (2.6 kg, [33]). When comparing males and females within our study, the percentage of breast fillet found in the senior laying hens was similar to that of the NH × Bresse and Bresse × WR males, and even slightly higher in LH and LH × WR, which underlines the balanced performance profiles in these breeds for males and females. 

### 4.6. Animal Welfare of the Hens

One of the welfare issues of laying hens in both conventional and organic husbandry systems is the appearance of keel bone deformations [34]. There are several factors contributing to the development of keel bone deformations, among which are high productivity, age at first lay, and inactivity of the layers [35]. While inactivity is not a likely cause in organic husbandry, with the required outdoor run and the supply of enrichment material, high productivity and age at first lay are as relevant as in conventional egg production. Although the laying performance of the dual-purpose hens studied here was much lower than that in highly productive layer hybrids, we still found a considerable frequency of keel bone deformations. A closer look at the differences between the breeds at the age of 50 weeks reveals that LH hens had a significantly higher frequency of intact keel bones, while the other breeds did not differ. As discussed previously, LH hens also started laying later and had the lowest laying performance of all breeds, while the others did not differ from each other. At the age of 72 weeks, LH hens had the highest frequency of hens that were not laying, based on palpation of the pelvic bones. It can therefore be concluded that the generally lower laying performance of the tested LH hens was associated with a lower frequency of keel bone deformations.

### 4.7. Sibling Performance

Due to their balanced performance profile, dual-purpose chickens are inherently less efficient in converting feed to food than high-producing layer hybrids and broilers. Still, within dual-purpose breeds, the question of efficiency and where the balance between eggs and meat lies remains highly relevant in view of a sustainable food production. In a study on life cycle assessments of intensive chicken production, Heines et al. [36] found that laying hens converted protein better than broilers, when protein quality was considered. In agreement with Heines et al. [36], we found an FCR for egg production of 2.78–2.92 kg feed kg^−1^ egg mass for LH × WR, NH × Bresse, and Bresse × WR, while the FCR during mixed-sex rearing was considerably higher, with 4.0–4.1 kg feed kg^−1^ weight gain until the age of 18 weeks. The main reason for FCR being higher for rearing was that the feeding regime focused on the pullets rather than maximum growth (although energy concentration was higher than planned, as discussed above). Moreover, FCR increases with the duration of the fattening period due to the maintenance requirements of the birds, so the extended fattening period of 18 weeks added to the difference. The efficiency of a dual-purpose breed can only be judged when the performance of both sexes is seen together. When expressing the efficiency of a pair of chickens (one male and one female) as kg feed kg^−1^ marketable product, we found a very similar FCR of 3.69–3.77 for LH × WR, NH × Bresse, and Bresse × WR, while purebred LH was less efficient with an FCR of 6.35 kg feed kg^−1^ marketable product. During rearing, LH did not differ from the other breeds, so the lower laying performance of LH clearly decreased their overall efficiency in converting feed to food. These observations allow the conclusion that crossing the breed LH with a layer breed not only improved laying performance but also the overall efficiency of the birds. From a sustainability standpoint, the challenge when keeping LH or other dual-purpose breeds with a focus on meat lies in reducing feed costs and feed losses as much as possible, in order to improve their efficiency.

## 5. Conclusions

Crossing the breed LH with the layer breed WR was found to almost double the laying performance of the crossbred hens compared the purebreds, but did not affect the fattening performance of the males. The efficiency of the birds expressed as feed needed to produce marketable products was thereby greatly improved. Purebred LH males showed fewer pecking injuries on the combs than the other breeds, which is interpreted as a sign of less aggressive behavior. Due to the rearing feed in week 8 and after being higher in energy than planned, the males of the dual-purpose crosses NH × Bresse and Bresse × WR reached higher growth rates than found in a previous study. Because rearing was mixed-sex, the downside was the development of a fatty liver syndrome in the laying hens, which affected egg shell quality and resulted in a high proportion of non-marketable eggs. This observation underlines the importance of adjusting the feeding regime to the needs of the pullets when both sexes are reared together. Despite these difficulties, both the novel cross LH × WR and the already established dual-purpose crosses NH × Bresse and Bresse × WR showed a balanced performance profile between egg and meat production, with 242–250 eggs per hen alive and a final live weight of the males of 2924–3105 g after 18 weeks of rearing.

## Figures and Tables

**Table 1:** Number of hatching eggs, hatched chicks, and hatchability per genotype.

	LH	LH × WR	NH × Bresse	Bresse × WR
Hatching eggs	392	404	330	350
Chicks	278	226	203	178
Hatchability, %	71	56	62	51

**Table 2:** Analyzed nutrient composition of the feed mixtures, g kg^−1^ (as fed) unless stated otherwise.

	Starter 1 Week 1–3	Starter 2 Week 4–7	Pullet 1 Week 8–11	Pullet 2 ^2^ Week 12–18	Layer 1 Week 20–67	Layer 2 Week 68–72	Oat Grains Week 20–72
Crude protein	211	171	194	167	159	157	100
Ether extracts	72	67	67	57	59	52	37
Crude fiber	63	71	53	68	71	72	104
Starch	311	386	379	391	340	319	372
Sugar	39	34	42	36	32	36	9
MJ AME_N_^1^	11.5	11.8	12.2	11.6	10.6	10.0	9.2
Lysine	10.1	7.6	10.2	8.2	7.2	6.9	4.4
Methionine	3.9	3.1	3.4	2.9	2.6	2.6	1.8
Cystine	3.5	3.0	3.4	3.0	2.8	2.6	3.1
g methionine MJ^−1^ AME_N_^1^	0.34	0.26	0.28	0.25	0.25	0.26	0.20
Calcium	15.8	7.0	11.0	7.2	34	41	
Phosphorus	10.7	6.2	9.7	6.3	7.2	6.4	

^1^ AME_N_ = apparent metabolizable nitrogen-corrected energy, calculated according to WPSA (1984); ^2^ in week 19, a 50:50 mixture of Pullet 2 and Layer 1 was fed.

**Table 3:** Feed consumption during mixed-sex rearing, and fattening and slaughter performance of the males of dual-purpose breeds (GT; LH = Deutsches Lachshuhn WR = White Rock, NH = New Hampshire), lsmeans of the effect of breed except for life weight (lsmeans of the interaction GT × age), mean values, and standard deviation for males’ valuable cuts.

	Genotype					*p* Values		
	LH	LH × WR	NH × Bresse	Bresse × WR	SEM ^1^	GT	Age	GT × Age
Feed consumption of mixed-sex birds, g day^−1^								
Week 1–15	76	72	80	76	3.34–3.57	0.533	<0.001	0.822
Week 1–18	84	78	85	83	3.70–3.82	0.344	<0.001	0.735
Feed conversion ratio of mixed-sex birds, kg feed kg^−1^ weight gain								
Week 1–15	3.6	3.5	3.6	3.5	0.13	0.868	<0.001	0.295
Week 1–18	4.1	4.0	4.1	4.1	0.12–0.13	0.958	<0.001	0.459
Males	N = 86/56	N = 91/56	N = 89/59	N = 90/60				
Live weight week 15, g	2430 ^a^	2439 ^a^	2717 ^c^	2629 ^b^	21.4–22.3	<0.001	<0.001	<0.001
Live weight week 18, g	2944 ^a^	2924 ^a^	3105 ^b^	3075 ^b^	22.7–23.0			
DWG week 1–15, g	22.8 ^a^	22.8 ^a^	25.5 ^c^	24.7 ^b^	0.19–0.22	<0.001	<0.001	<0.001
DWG week 1–18, g	22.8 ^a^	22.5 ^a^	24.4 ^b^	23.9 ^b^	0.19–0.21			
Carcass weight week 15, g	1341 ^a^	1367 ^a^	1623 ^c^	1498 ^b^	21.6–22.8	<0.001		
Carcass weight week18, g	1703 ^a^	1726 ^a^	1926 ^b^	1892 ^b^	20.7–23.4	<0.001		
Dressing percentage week 15, %	55.1 ^a^	55.9 ^a^	59.0 ^b^	58.4 ^b^	0.29–0.30	<0.001		
Dressing percentage week 18, %	58.4 ^a^	58.4 ^a^	61.9 ^c^	60.8 ^b^	0.25–0.28	<0.001		
	Mean ± SD ^2^	Mean ± SD	Mean ± SD	Mean ± SD				
Males’ valuable cuts	N = 3	N = 3	N = 3	N = 3				
Breast fillet week 15, %	15.1 ± 0.8	15.9 ± 1.1	17.6 ± 0.5	18.5 ± 1.2				
Breast fillet week 18, %	15.7 ± 1.4	15.6 ± 0.6	19.0 ± 0.9	17.5 ±0.9				
Legs week 15, %	39.0 ± 0.7	39.4 ± 0.9	39.5 ± 0.9	39.2 ± 1.4				
Legs week 18, %	39.4 ± 1.0	40.6 ± 0.2	42.9 ± 3.4	41.8 ± 0.1				
Females	N = 86	N = 88	N = 91	N = 83				
Live weight week 15, g	1786 ^ab^	1756 ^a^	1862 ^c^	1824 ^bc^	10.9–11.4	<0.001	<0.001	<0.001
Live weight week 18, g	2143 ^b^	2054 ^a^	2204 ^c^	2133 ^b^	10.9–11.3			

^1^ Standard error of the mean, if given as a range then because of uneven data distribution; least square means with no letter in common indicate significant differences. ^2^ Mean and standard deviation.

**Table 4:** Feed consumption during laying, and laying and slaughter performance of the hens of dual-purpose breeds (GT; LH = Deutsches Lachshuhn, WR = White Rock, NH = New Hampshire); lsmeans of the effect of breed except for life weight (lsmeans of the interaction GT × age), mean values and standard deviation for slaughter performance.

	Breed						*p* Values	
	LH	LH × WR	NH × Bresse	Bresse × WR	SEM ^1^	GT	Age	GT × Age
Feed consumption, g day^−1^								
Week 20–72	111 ^a^	122 ^b^	124 ^b^	125 ^b^	1.1	<0.001	<0.001	0.082
Laying performance and egg quality								
Total eggs, %	34.7 ^a^	68.8 ^b^	68.5 ^b^	66.4 ^b^	0.71	<0.001	<0.001	<0.001
Marketable eggs, %	9.7 ^a^	36.5 ^b^	41.7 ^b^	31.6 ^b^	0.73	<0.001	<0.001	<0.001
Egg weight, g	55.1 ^a^	62.7 ^b^	65.3 ^c^	66.5 ^d^	0.65–0.85	<0.001	<0.001	0.577
Egg shape index	74.0 ^a^	75.8 ^b^	74.9 ^ab^	75.8 ^b^	0.35–0.43	0.002	<0.001	0.241
Yolk, %	30.5 ^c^	28.3 ^b^	27.9 ^b^	26.3 ^a^	0.25–0.32	<0.001	<0.001	0.028
Albumen, %	59.8 ^a^	62.0 ^b^	63.0 ^bc^	63.9 ^c^	0.27–0.34	<0.001	<0.001	0.054
Yolk color	9.8 ^b^	9.3 ^a^	9.2 ^a^	9.4 ^ab^	0.12–0.15	0.006	<0.001	0.030
Shell, %	9.5 ^b^	9.6 ^b^	9.0 ^a^	9.6 ^b^	0.07–0.09	<0.001	<0.001	0.847
Live weight, g	N = 83	N = 82	N = 84	N = 81				
Week 32	2837 ^c^	2520 ^b^	2568 ^b^	2343 ^a^	23.3–24.0	<0.001	<0.001	<0.001
Week 50	2812 ^c^	2501 ^ab^	2609 ^b^	2409 ^a^	23.3–23.9	<0.001	<0.001	
Week 72	2996 ^c^	2571 ^a^	2726 ^b^	2509 ^a^	23.1–24.3	<0.001	<0.001	
	Mean ± SD ^2^	Mean ± SD	Mean ± SD	Mean ± SD				
Slaughter performance	N = 3	N = 2	N = 3	N = 3				
Live weight, g	3166 ± 121	2558 ± 161	2613± 157	2428± 132				
Carcass weight, g	2037± 186	1528 ±46	1515 ± 131	1339± 122				
Dressing percentage, %	64.2 ± 3.4	57.6 ± 0.7	57.9 ± 1.6	55.0 ± 2.5				
Breast fillet, %	17.9 ± 1.0	16.9 ± 0.2	18.8 ± 0.4	18.4 ± 1.0				
Legs, %	30.0 ± 3.1	33.6± 0.4	33.7 ± 0.7	33.5 ± 1.2				

^1^ Standard error of the mean, if given as a range then because of uneven data distribution. ^2^ Mean and standard deviation; least square means with no letter in common indicate significant differences.

**Table 5:** Fattening and laying performance of dual-purpose breeds and their efficiency, expressed as the arithmetic means of feed consumption and production of marketable products (carcasses ready for sale, eggs in shell) of a sibling pair of the respective breed (LH = Deutsches Lachshuhn, WR = White Rock, NH = New Hampshire).

	Breed			
	LH	LH × WR	NH × Bresse	Bresse × WR
Feed consumption				
Week 1–18, kg bird^−1^	10.6	9.9	10.8	10.5
Week 19, kg pullet^−1^	1.72	1.54	1.60	1.64
Week 20–72, kg hen^−1^	44.4	48.8	49.9	50.2
Total, kg per sibling pair	67.3	70.1	73.1	72.8
Fattening and laying performance				
Carcass weight males week 18, kg	1.70	1.73	1.93	1.89
Total egg mass, kg hen^−1^	6.9	15.7	16.3	16.1
Carcass weight senior laying hen, kg	2.04	1.53	1.52	1.34
Total produced food	10.6	19.0	19.5	19.3
Feed conversion ratio, kg feed kg^−1^ marketable product				
Week 1–72	6.35	3.69	3.75	3.77

## Data Availability

The data presented in this study are available on request from the corresponding author.

## Supplementary Materials

The following supporting information can be downloaded at: https://www.mdpi.com/article/10.3390/ani13192999/s1, Table S1: Description of the data set: Animal numbers, feed consumption during rearing and laying, and slaughter and laying performance of dual-purpose breeds; Table S2: Welfare indicators of the males of dual-purpose breeds, % of birds with the respective score; Table S3: Welfare indicators of the hens of dual-purpose breeds, % of birds with the respective score; Figure S1: Laying performance (% per hen alive) during the second laying period of the hens of the dual-purpose cross LH × WR (=Caramel) which were kept after the end of the study.
