# Supplementary material for: Crossing the Old Local Breed Deutsches Lachshuhn with the Layer Breed White Rock: Effects on Laying Performance of the Females and Fattening Performance of the Males
Source: Animals (Basel). 2023 Sep 22;13(19):2999. doi: 10.3390/ani13192999 (PMC10571803; doi:10.3390/ani13192999)
Supplement: Supplementary file 1 [file animals-13-02999-s001.zip › animals-2575129-supplementary.pdf]

**Supplemental Material:**

**Table S1.** Description of the data set: Animal numbers, feed consumption during rearing and laying, and slaughter and laying performance of dual-purpose genotypes (LH = Deutsches Lachshuhn, WR = White Rock, NH= New Hampshire, Bresse = Bresse Gauloise), means ± standard deviation

| | Genotype | | | |
|---|---|---|---|---|
| | **LH** | **LH × WR** | **NH × Bresse** | **Bresse × WR** |
| **Rearing, week 1-18** | | | | |
| Number of groups | 2 | 2 | 2 | 2 |
| Number of day-old chicks | 278 | 226 | 203 | 178 |
| Feed consumption, g $day^{-1}$ | 84 ± 39.2 | 78 ± 36.0 | 85 ± 37.5 | 83 ± 36.4 |
| Number of males at 3 weeks of age | 83 | 91 | 90 | 92 |
| Daily weight gain of males, g | 20 ± 6.6 | 19 ± 6.4 | 21 ± 7.1 | 20 ± 6.6 |
| Final live weight of males, g | 2465 ± 431 | 2405 ± 466 | 2565 ± 480 | 2533 ± 505 |
| Carcass weight of males, g | 1713 ± 249 | 1690 ± 242 | 1776 ± 244 | 1769 ± 240 |
| **Laying, week 20-72** | | | | |
| Number of groups | 2 | 2 | 2 | 2 |
| Number of layers at 20 weeks of age | 83 | 82 | 84 | 81 |
| Feed consumption, g $day^{-1}$ | 111 ± 20 | 122 ± 18 | 124 ± 14 | 125 ± 16 |
| Laying performance, % per hen alive | 35 ± 19 | 69 ± 20 | 69 ± 15 | 66 ± 18 |
| Live weight of layers week 18, g | 2151 ± 116 | 2054 ± 161 | 2204 ± 174 | 2133 ± 170 |
| Live weight of layers week 72, g | 2984 ± 245 | 2575 ± 275 | 2729 ± 254 | 2514 ± 192 |

**Table S2**. Welfare indicators of the males of dual-purpose genotypes, % of birds with the respective score, with score "0" indicating an unimpaired state, "1" minor changes and "2" major damages (LH= Deutsches Lachshuhn, WR = White Rock, NH= New Hampshire, Bresse = Bresse Gauloise; columns with no letter in common indicate significant differences)

| | LH | | | LH × WR | | | NH × Bresse | | | Bresse × WR | | | P value |
|---|---|---|---|---|---|---|---|---|---|---|---|---|---|
| | **0** | **1** | **2** | **0** | **1** | **2** | **0** | **1** | **2** | **0** | **1** | **2** | |
| **Week 12** | *N = 86* | | | *N = 91* | | | *N = 89* | | | *N = 91* | | | |
| Pecking injuries on the comb | $86^{b}$ | 14 | 0 | $66^{a}$ | 33 | 1 | $65^{a}$ | 35 | 0 | $51^{a}$ | 45 | 4 | <0.001 |
| Completeness of plumage on the back | $36^{a}$ | 34 | 30 | $90^{b}$ | 10 | 0 | $82^{b}$ | 18 | 0 | $86^{b}$ | 14 | 0 | <0.001 |
| Cleanliness of plumage on the back | $98^{b}$ | 2 | 0 | $82^{a}$ | 14 | 3 | $71^{a}$ | 26 | 3 | $79^{a}$ | 19 | 2 | 0.002 |
| Pecking injuries on the back | 96 | 3 | 1 | 100 | 0 | 0 | 100 | 0 | 0 | 100 | 0 | 0 | 1.000 |
| Cleanliness of the cloaca | 97 | 3 | 0 | 93 | 7 | 0 | 97 | 3 | 0 | 96 | 4 | 0 | 0.714 |
| Foot pad lesions | 100 | 0 | 0 | 100 | 0 | 0 | 100 | 0 | 0 | 100 | 0 | 0 | |
| Hock burns | 100 | 0 | 0 | 100 | 0 | 0 | 100 | 0 | 0 | 100 | 0 | 0 | |
| Toe damages | 99 | 1 | 0 | 99 | 1 | 0 | 100 | 0 | 0 | 100 | 0 | 0 | 1.000 |
| Breast blisters | 99 | 1 | 0 | 92 | 8 | 0 | 96 | 4 | 0 | 91 | 9 | 0 | 0.198 |
| **Week 15** | *N = 29* | | | *N = 30* | | | *N = 30* | | | *N = 30* | | | |
| Pecking injuries on the comb | 69 | 31 | 0 | 67 | 30 | 3 | 53 | 40 | 7 | 53 | 40 | 7 | 0.385 |
| Completeness of plumage on the back | $38^{a}$ | 52 | 10 | $90^{c}$ | 10 | 0 | $53^{ab}$ | 47 | 0 | $80^{bc}$ | 20 | 0 | <0.001 |
| Cleanliness of plumage on the back | $97^{b}$ | 3 | 0 | $100^{b}$ | 0 | 0 | $53^{a}$ | 40 | 7 | $70^{ab}$ | 23 | 7 | 0.005 |
| Pecking injuries on the back | 90 | 10 | 0 | 100 | 0 | 0 | 100 | 0 | 0 | 100 | 0 | 0 | 1.000 |
| Cleanliness of the cloaca | 100 | 0 | 0 | 100 | 0 | 0 | 100 | 0 | 0 | 100 | 0 | 0 | |
| Foot pad lesions | 100 | 0 | 0 | 100 | 0 | 0 | 100 | 0 | 0 | 97 | 3 | 0 | 1.000 |
| Hock burns | 100 | 0 | 0 | 100 | 0 | 0 | 100 | 0 | 0 | 100 | 0 | 0 | |
| Toe damages | 100 | 0 | 0 | 97 | 3 | 0 | 100 | 0 | 0 | 100 | 0 | 0 | 1.000 |
| Breast blisters | 90 | 10 | 0 | 90 | 10 | 0 | 97 | 3 | 0 | 83 | 13 | 3 | 0.447 |
| **Week 18** | *N = 56* | | | *N = 56* | | | *N = 59* | | | *N = 60* | | | |
| Pecking injuries on the comb | $70^{b}$ | 27 | 4 | $64^{b}$ | 30 | 5 | $29^{a}$ | 59 | 12 | $35^{a}$ | 42 | 23 | <0.001 |
| Completeness of plumage on the back | $54^{a}$ | 46 | 0 | $89^{b}$ | 11 | 0 | $66^{a}$ | 34 | 0 | $78^{b}$ | 22 | 0 | 0.001 |
| Cleanliness of plumage on the back | $78^{b}$ | 20 | 2 | $64^{ab}$ | 30 | 5 | $49^{a}$ | 34 | 17 | $63^{ab}$ | 32 | 5 | 0.006 |
| Pecking injuries on the back | 100 | 0 | 0 | 91 | 9 | 0 | 100 | 0 | 0 | 98 | 2 | 0 | 0.478 |
| Cleanliness of the cloaca | $68^{a}$ | 32 | 0 | $93^{b}$ | 7 | 0 | $86^{ab}$ | 12 | 2 | $88^{ab}$ | 12 | 0 | 0.005 |
| Foot pad lesions | 100 | 0 | 0 | 100 | 0 | 0 | 100 | 0 | 0 | 100 | 0 | 0 | |
| Hock burns | 100 | 0 | 0 | 100 | 0 | 0 | 100 | 0 | 0 | 100 | 0 | 0 | |
| Toe damages | 98 | 2 | 0 | 100 | 0 | 0 | 98 | 0 | 2 | 98 | 2 | 0 | 1.000 |
| Breast blisters | 75 | 18 | 7 | 86 | 9 | 5 | 86 | 7 | 7 | 85 | 12 | 3 | 0.366 |

**Table S3**. Welfare indicators of the hens of dual-purpose genotypes, % of birds with the respective score, with score "0" indicating an unimpaired state, "1" minor changes and "2" major damages, except for laying activity, where "0" = yes, "1" = no, "2" = maybe (LH = Deutsches Lachshuhn, WR = White Rock, NH= New Hampshire, Bresse = Bresse Gauloise; columns with no letter in common indicate significant differences)

| | LH | | | LH × WR | | | NH × Bresse | | | Bresse × WR | | | P value |
|---|---|---|---|---|---|---|---|---|---|---|---|---|---|
| | **0** | **1** | **2** | **0** | **1** | **2** | **0** | **1** | **2** | **0** | **1** | **2** | |
| **Week 18** | *N = 83* | | | *N = 82* | | | *N = 84* | | | *N = 81* | | | |
| Pecking injuries on the comb | 95[a] | 5 | 0 | 79[b] | 21 | 0 | 69b[c] | 30 | 1 | 62[c] | 35 | 4 | <0.001 |
| Completeness of plumage on the back | 87 | 13 | 0 | 100 | 0 | 0 | 100 | 0 | 0 | 100 | 0 | 0 | 1.000 |
| Cleanliness of plumage on the back | 70[b] | 29 | 1 | 87[a] | 13 | 0 | 57[bc] | 39 | 4 | 44[c] | 49 | 6 | <0.001 |
| Pecking injuries on the back | 100 | 0 | 0 | 100 | 0 | 0 | 100 | 0 | 0 | 100 | 0 | 0 | |
| Complete plumage around cloaca | 100 | 0 | 0 | 100 | 0 | 0 | 100 | 0 | 0 | 100 | 0 | 0 | |
| Cleanliness of the cloaca | 86[ab] | 14 | 0 | 99[a] | 1 | 0 | 82[b] | 17 | 1 | 78[b] | 22 | 0 | 0.022 |
| Pecking injuries around the cloaca | 100 | 0 | 0 | 100 | 0 | 0 | 99 | 1 | 0 | 100 | 0 | 0 | 1.000 |
| Foot pad lesions | 100 | 0 | 0 | 100 | 0 | 0 | 100 | 0 | 0 | 100 | 0 | 0 | |
| Toe damages | 95 | 5 | 0 | 100 | 0 | 0 | 100 | 0 | 0 | 99 | 1 | 0 | 0.674 |
| Keelbone deformations | 96[a] | 4 | 0 | 94[ab] | 6 | 0 | 86[ab] | 14 | 0 | 81[b] | 19 | 0 | 0.013 |
| Laying activity | 0 | 96 | 4 | 2 | 91 | 7 | 18 | 63 | 19 | 23 | 63 | 14 | 0.155 |
| **Week 50** | *N = 84* | | | *N = 79* | | | *N = 82* | | | *N = 79* | | | |
| Pecking injuries on the comb | 67[a] | 31 | 2 | 64[b] | 33 | 3 | 41[bc] | 56 | 2 | 25[c] | 66 | 9 | <0.001 |
| Completeness of plumage on the back | 15[c] | 44 | 41 | 28[bc] | 42 | 30 | 40[a] | 54 | 6 | 46[ab] | 34 | 20 | <0.001 |
| Cleanliness of plumage on the back | 74 | 26 | 0 | 84 | 16 | 0 | 78 | 22 | 0 | 77 | 23 | 0 | 0.473 |
| Pecking injuries on the back | 88 | 12 | 0 | 87 | 13 | 0 | 88 | 12 | 0 | 76 | 24 | 0 | 0.083 |
| Complete plumage around cloaca | 95[a] | 5 | 0 | 97[a] | 3 | 0 | 97[a] | 2 | 1 | 43[b] | 22 | 35 | <0.001 |
| Cleanliness of the cloaca | 36[b] | 59 | 5 | 44[b] | 56 | 0 | 41[b] | 59 | 0 | 68[a] | 32 | 0 | <0.001 |
| Pecking injuries around the cloaca | 99[a] | 0 | 1 | 99[a] | 1 | 0 | 99[a] | 0 | 1 | 77[b] | 23 | 0 | <0.001 |
| Foot pad lesions | 98 | 2 | 0 | 89 | 11 | 0 | 92 | 8 | 0 | 92 | 8 | 0 | 0.236 |
| Toe damages | 100 | 0 | 0 | 100 | 0 | 0 | 100 | 0 | 0 | 99 | 1 | 0 | 1.000 |
| Keelbone deformations | 78[a] | 18 | 4 | 58[b] | 37 | 5 | 46[b] | 42 | 12 | 54[b] | 38 | 8 | <0.001 |
| Laying activity | 88[ab] | 1 | 11 | 95[ab] | 0 | 5 | 81[b] | 1 | 18 | 98[a] | 0 | 2 | 0.005 |
| **Week 72** | *N = 76* | | | *N = 76* | | | *N = 79* | | | *N = 71* | | | |
| Pecking injuries on the comb | 80[a] | 16 | 4 | 58[b] | 39 | 3 | 44[bc] | 47 | 9 | 37[c] | 52 | 11 | <0.001 |
| Completeness of plumage on the back | 87[a] | 4 | 9 | 11[c] | 29 | 61 | 33[b] | 53 | 14 | 46[b] | 31 | 23 | <0.001 |
| Cleanliness of plumage on the back | 71[a] | 29 | 0 | 43[b] | 53 | 4 | 58[ab] | 39 | 3 | 15[b] | 75 | 10 | <0.001 |
| Pecking injuries on the back | 99 | 1 | 0 | 97 | 3 | 0 | 97 | 3 | 0 | 93 | 7 | 0 | 0.300 |
| Complete plumage around cloaca | 100 | 0 | 0 | 100 | 0 | 0 | 96 | 4 | 0 | 38 | 20 | 42 | 1.000 |

| | | | | | | | | | | | | |
|---|---|---|---|---|---|---|---|---|---|---|---|---|
| Cleanliness of the cloaca | 40 | 43 | 17 | 37 | 53 | 10 | 53 | 35 | 11 | 67 | 30 | 3 | <0.001 |
| Pecking injuries around the cloaca | 100 | 0 | 0 | 100 | 0 | 0 | 96 | 4 | 0 | 86 | 11 | 3 | 1.000 |
| Foot pad lesions | 97[a] | 3 | 0 | 83[b] | 17 | 0 | 82[b] | 18 | 0 | 77[b] | 23 | 0 | 0.027 |
| Toe damages | 100 | 0 | 0 | 100 | 0 | 0 | 100 | 0 | 0 | 100 | 0 | 0 | |
| Keelbone deformations | 84 | 13 | 3 | 72 | 20 | 8 | 39 | 41 | 20 | 56 | 30 | 14 | 0.071 |
| Laying activity | 58[b] | 21 | 21 | 78[a] | 11 | 11 | 70[ab] | 8 | 22 | 86[a] | 10 | 4 | 0.004 |

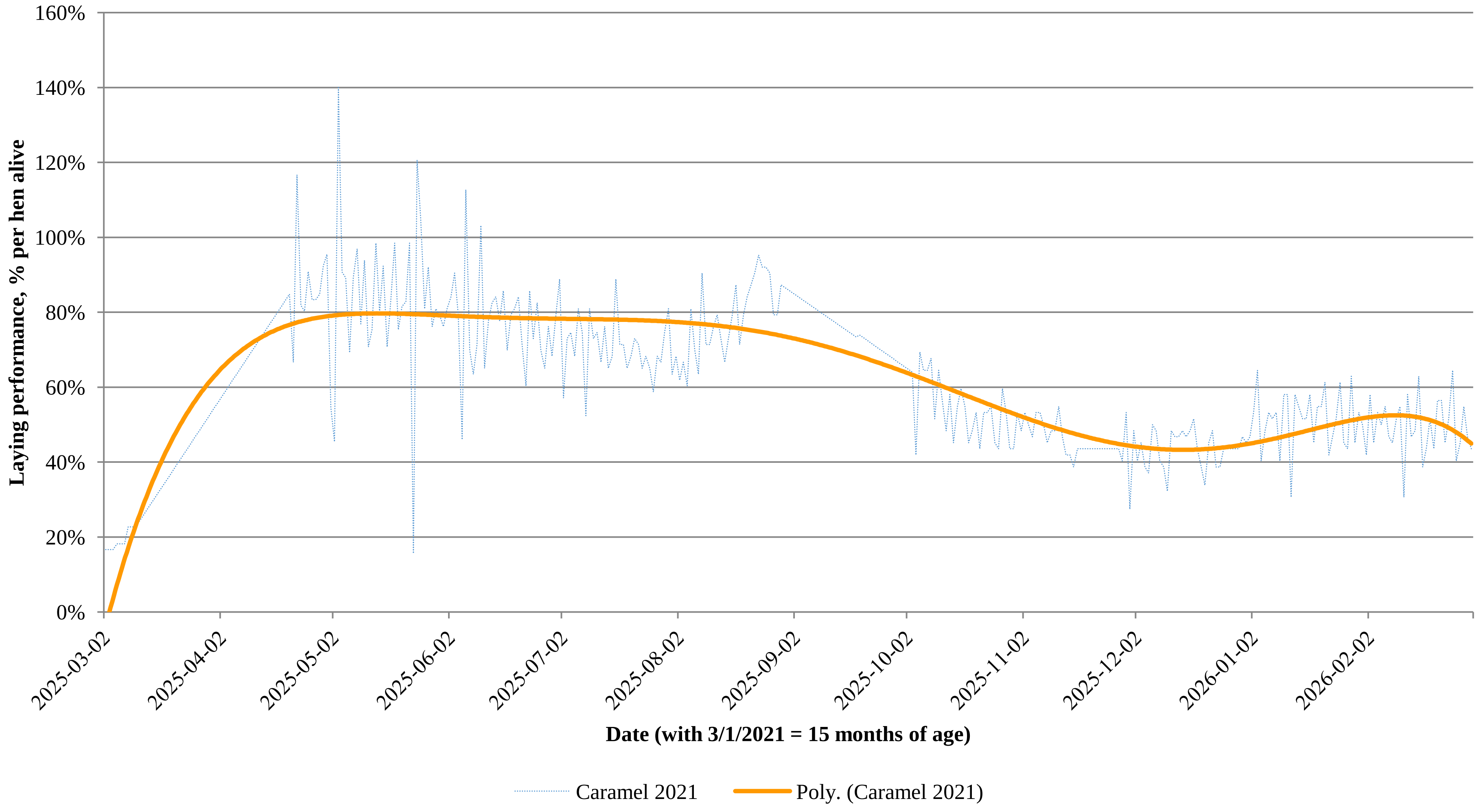


**Figure S1**. Laying performance (% per hen alive) during the second laying period of the the hens of the dual-purpose cross LH × WR (= Caramel) which were kept after the end of the study.
